# Thermally Stable and Nonflammable Electrolytes for Lithium Metal Batteries: Progress and Perspectives

**DOI:** 10.1002/smsc.202100058

**Published:** 2021-08-21

**Authors:** Qian-Kui Zhang, Xue-Qiang Zhang, Hong Yuan, Jia-Qi Huang

**Affiliations:** ^1^ School of Materials Science and Engineering Beijing Institute of Technology Beijing 100081 China; ^2^ Advanced Research Institute of Multidisciplinary Science Beijing Institute of Technology Beijing 100081 China; ^3^ Beijing Key Laboratory of Green Chemical Reaction Engineering and Technology Department of Chemical Engineering Tsinghua University Beijing 100084 China

**Keywords:** battery safety, lithium metal batteries, nonflammable electrolytes, thermal stability of electrolytes

## Abstract

Lithium (Li) metal battery is considered as a promising next‐generation high‐energy‐density battery system. Battery safety is a foundation for the practical applications of Li metal batteries. In addition to uncontrollable Li plating/stripping, however, the employment of solvents and Li salts with poor thermal stability and high flammability also induces potential safety hazards to Li metal batteries based on liquid electrolytes, which emerges as a challenging but uncompromising task for Li metal batteries. Therefore, it is of great significance to focus and improve the thermal stability and nonflammability of liquid electrolytes while maintaining its role in regulating the uniformity of Li deposition. Herein, the possible thermal runaway mechanism of Li metal batteries is first discussed, especially the difference from the thermal runaway mechanism of Li‐ion batteries. Second, the measurement methods of thermal stability and nonflammability of liquid electrolytes are summarized, including the basic principles and application examples. Third, recent progress of nonflammable electrolytes for Li metal batteries is reviewed according to the types of electrolyte. Finally, the perspectives of further designing thermally stable and nonflammable electrolytes for Li metal batteries are presented.

## Graphical abstract

High safety is the foundation of practical applications of lithium (Li) metal batteries. Herein, the thermal runaway mechanism of Li metal batteries is prospectively analyzed. The measurement methods and research progress of thermal stability and nonflammability of liquid electrolytes are summarized. Furthermore, the perspectives of designing thermally stable and nonflammable electrolytes for Li metal batteries are presented.

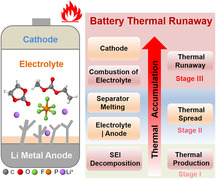

## 1 Introduction

In the pursuit of decarbonization and wireless society, high‐energy‐density secondary batteries are imperative and have attracted much interest around the world.^[^ 1, 2, 3, 4 ^]^ The increasing demand of electric vehicles, portable electronic products, and large‐scale grids has promoted intensive research on high‐energy‐density secondary batteries.^[^ 5, 6, 7, 8 ^]^ Lithium metal battery (LMB) is a promising high‐energy‐density battery system with a practical specific energy over 350 Wh kg^−1^ because lithium metal anode has a high theoretical specific capacity (3860 mAh g^−1^) and a low electrode potential (−3.04 V vs standard hydrogen electrode).^[^ 9, 10, 11, 12 ^]^ Generally, when lithium metal anodes are used to replace graphite anodes, the specific energy of lithium metal anode can increase by 40–50% compared with that of lithium‐ion batteries (LIBs).^[^ 13, 14, 15, 16 ^]^ In terms of the type of LMBs, intercalation cathodes have unique advantages to match with lithium metal anodes due to the technological maturity and the compatibility with current LIB manufacturing compared with conversion cathodes, such as oxygen and sulfur cathodes.^[^ 17, 18, 19, 20, 21 ^]^ By integrating a lithium metal anode with a lithium‐rich or nickel‐rich layered oxide cathode, the practical specific energy of LMBs with liquid electrolytes can achieve more than 350 Wh kg^−1^, becoming a promising high‐energy‐density battery system in the near future.^[^ 22, 23, 24, 25 ^]^

High safety is the prerequisite for the practical applications of LMBs. In 1985, Moli Energy, a Canada company, produced Li/MoS_2_ batteries (AA batteries, ≈100 Wh kg^−1^) for the first time and put them on the market as the power of consumer electronics.^[^ ^26^ ^]^ However, Li/MoS_2_ batteries suffer safety issues, such as fire and explosion, when in use. Moli energy had to recall all batteries, and the first attempt toward the commercialization of LMBs fails. The safety issues of LMBs are directly related to the overgrown lithium dendrites and highly flammable liquid electrolytes.^[^ 27, 28, 29 ^]^ In nature, the formation of lithium dendrites is primarily induced by liquid electrolytes. Consequently, liquid electrolytes dictate not only the life span but also the safety degree of LMBs. LMBs are revived after 2010 due to the strong demand of high‐energy‐density batteries and the emerging materials and technologies to solve the inherent problems.^[^ ^30^ ^]^ New electrolyte design,^[^ 31, 32, 33, 34, 35 ^]^ composite lithium anode with a 3D host, ^[^ 36, 37, 38 ^]^ artificial coating,^[^ 39, 40, 41, 42 ^]^ and theoretical simulations^[^ ^43^ ^]^ have been intensively investigated to suppress the formation of lithium dendrites and remarkable advances have been achieved in prolonging the cycle life of LMBs as summarized in recent impactful reviews.^[^ 44, 45 ^]^ However, in addition to suppress lithium dendrites, the improvement of the thermal stability and nonflammability of liquid electrolytes also deserves great attention regarding the safety of LMBs.^[^ 46, 47, 48, 49, 50, 51, 52 ^]^

The current LMBs generally use liquid electrolytes consisting of thermally unstable and flammable organic solvents (e.g., carbonates and ethers) and thermally unstable salts (e.g., lithium hexafluorophosphate, LiPF_6_), which is the root reason of the safety issues of liquid electrolytes.^[^ 53, 54 ^]^ Commonly used organic solvents include ethylene carbonate (EC), propylene carbonate (PC), ethyl methyl carbonate (EMC), diethyl carbonate (DEC), dimethyl carbonate (DMC), 1,3‐dioxalane (DOL), and 1,2‐dimethoxyethane (DME) as summarized in **Table** **1**. These solvents are generally with a low flash point, becoming the potential safety hazards of LMBs, just like the safety issues of LIBs. When the internal temperature in batteries increases due to the misuse or abuse at initial stage, the decomposition of organic solvents easily occurs, then accelerates the increase in temperature, and finally leads to the thermal runaway of batteries. When batteries are cracked, the expose of oxygen will ignite the flammable solvents, leading to severe fire and even explosion. In addition, when LiPF_6_ is introduced into organic solvents, the thermal instability of electrolytes aggravates. When the internal temperature in battery rises, LiPF_6_ undergoes thermal decomposition reactions to form phosphorus pentafluoride (PF_5_) before the decompositions of solvents. PF_5_ is a strong Lewis acid and has high reactivity with organic solvents, thereby giving rise to substantial decompositions of electrolytes easily.^[^ 55, 56, 57, 58 ^]^ Therefore, designing electrolytes with excellent thermal stability and nonflammability is critical for LMBs with high safety.

**Table 1:** Physical properties of common solvents

Solvent	Boiling point [°C]	Flashing point [°C]	Dielectric constant [ε]	Density [g cm^−3^]
1,2‐Dimethoxyethane (DME)	83	1.0	7.2	0.86
1,3‐Dioxalane (DOL)	74	−6.0	7.1	1.06
Ethylene carbonate (EC)	238	160	89.8	1.32
Propylene carbonate (PC)	242	132	66.1	1.20
Dimethyl carbonate (DMC)	90	17	3.1	1.06
Ethyl‐methylcarbonat (EMC)	108	27	2.4	1.01
Diethyl carbonate (DEC)	127	25	2.8	0.97
Fluoroethylene carbonate (FEC)	249	120	79.7	1.48
Bis(2,2,2‐trifluoroethyl) ether (BTFE)	64	1.7	/	1.40
Trimethyl phosphate (TMP)	197	107	20.7	1.21
Triethyl phosphate (TEP)	220	117	13.0	1.07

Compared with graphite‐based LIBs, the safety problems of LMBs are more complex and challenging due to the overgrowth of lithium dendrites and the generation of dead lithium.^[^ 59, 60, 61, 62, 63 ^]^ On the one hand, the growth of lithium dendrites can possibly pierce the separator, causing a short circuit in battery and quickly generating a large amount of reaction heat to induce the decomposition of electrolytes. On the other hand, high‐surface‐area dead lithium, which losses the electronic contact with current collectors, inevitably forms on the surface of lithium metal anode during cycles. Generally, these dead lithium are metallic lithium wrapped by solid electrolyte interphase (SEI). If the SEI on dead lithium surface is broken, metallic lithium will directly react with electrolytes to generate reaction heat. Due to the high specific surface area of dead lithium, the reaction heat cannot be overlooked, which contributes to the heat accumulation and temperature increase to induce the following decomposition of electrolyte at initial stage of thermal runaway. Taking the high reactivity of lithium metal anode and the instability of SEI into consideration, the temperature start point (denoted as *T*_1_ generally) toward thermal runaway may come earlier than that of graphite anode. The existence of dead lithium is a unique problem in LMBs, which is not involved in LIBs. Therefore, the design of electrolytes with high thermal stability and nonflammability in LMBs is more challenged than that of LIBs.

In this review, the possible thermal runaway mechanism of LMBs is first discussed, especially the difference from the thermal runaway mechanism of LIBs. Second, the measurement methods of thermal stability and flammability of liquid electrolytes are summarized, including the basic principles and application examples. Third, recent progress on thermal stable and nonflammable electrolytes in LMBs is reviewed according to the types of electrolyte. Finally, the perspectives of further designing thermal stable and nonflammable electrolytes for LMBs are presented.

## 2 Role of Electrolyte in Battery Thermal Runaway

The understanding of the thermal runaway mechanism is essential to circumvent the potential safety hazards of the battery. At present, the research of the thermal runaway of LMBs is significantly insufficient. However, the working mechanisms of LMBs with intercalation cathodes resemble that of LIBs except for the employment of lithium metal anode. Therefore, the thermal runaway mechanism of LIBs can provide many references to analyze and disclose that of LMBs.

**Figure 1:**
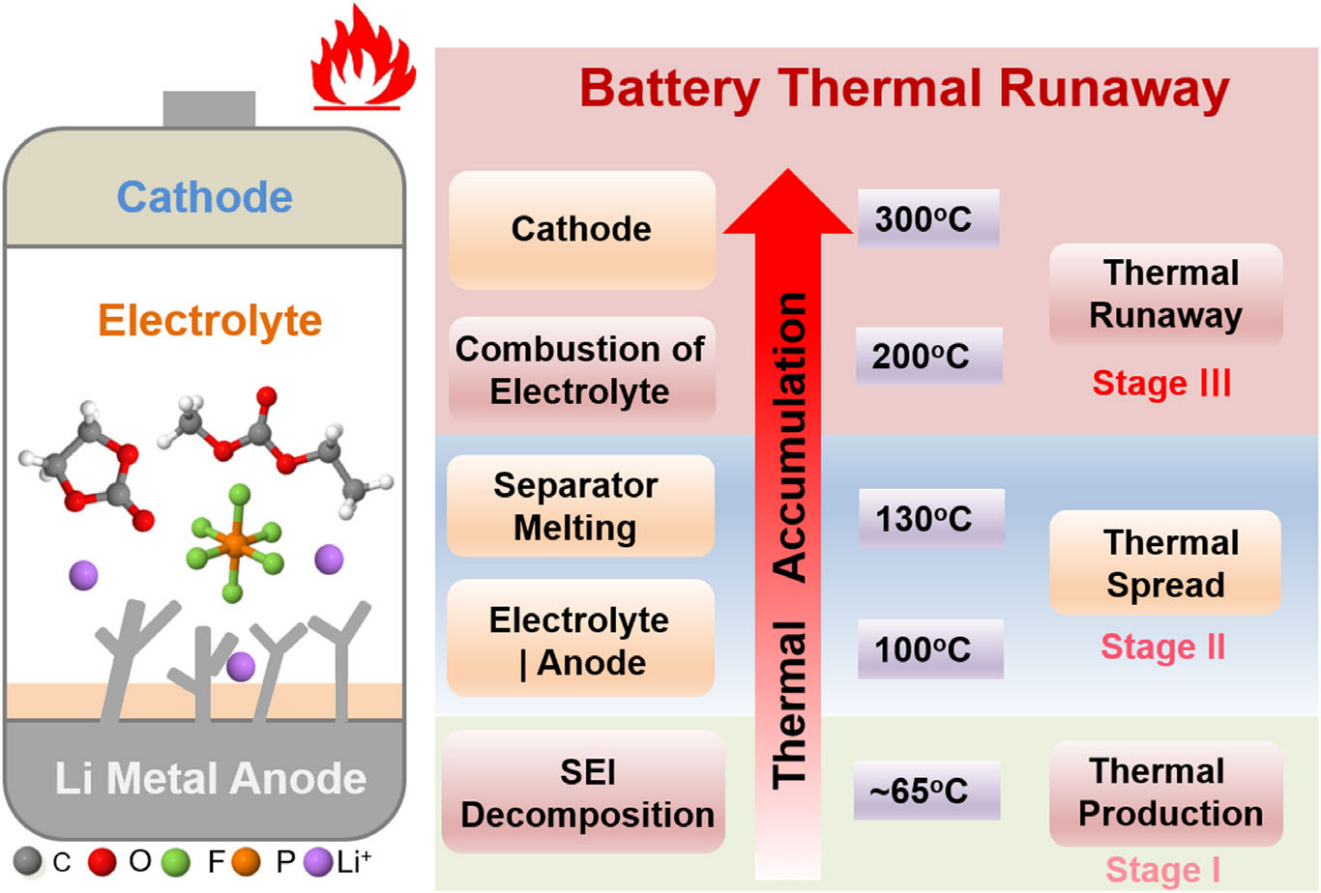
Schematic of thermal runaway process in LMBs.

When the battery is misused and abused by conditions such as thermal (e.g., exposure to high temperature), electrical (e.g., short circuit or overcharging), or mechanical (e.g., collision or puncture) conditions, heat accumulation will occur and lead to the rise of internal temperature of battery, possibly followed by a series of continuous exothermic processes, finally inducing battery thermal runaway (**Figure** **1**). The thermal runaway process can be mainly categorized into three stages: thermal production, thermal spread, and thermal runaway. When the internal temperature of battery increases to ≈65 °C, the thermal decomposition of SEI on anode occurs easily, which will release about 800 kJ kg^−1^_cell_ heat.^[^ 50, 64 ^]^ Then, the anode without SEI will continue to have parasitic reactions with electrolytes, constantly releasing reaction heat and cause the internal temperature of battery to be over 100 °C (Stage I: thermal production). Especially in electrolytes containing LiPF_6_, the decomposition reactions of organic solvents are more serious because the thermal decomposition product (PF_5_) of LiPF_6_ has high reactivity with organic solvents. When the internal temperature rises to 120–130 °C, the separator, generally polypropylene and/or polyethylene, is heated to shrink, which will cause the direct contact of anode and cathode, that is an internal short circuit in the battery. The internal short circuit in the batteries will produce much more heat according to Joule's law (Stage II: thermal spread). When the temperature rises over 200 °C (Stage III: thermal runaway), the thermal decomposition of solvents will form a large amount of hydrogen (H·) and hydroxide radicals (OH·), which will trigger a combustion reaction of electrolytes. Finally, when the temperature continues to rise to about 300 °C, the cathode (such as nickel‐rich layered oxide cathode) begins to decompose and produce more oxygen and combustible gas. The successive exothermic reactions will cause the internal pressure and temperature of a battery to rise sharply. When the internal pressure exceeds the limiting pressure of battery case, battery will crack and lots of oxygen meets flammable solvents, leading to severe safety accidents and disasters.

Compared with LIBs, LMBs also have unique aspects in terms of the thermal runaway mechanism. Lithium metal is more reactive than graphite and the reactions between lithium metal and electrolytes occur more easily. Moreover, Li deposition is generally nonuniform and even dendritic with high surface area. Li dendrites may pierce the separator and induce an internal short circuit of batteries, which directly initiates the thermal runaway of batteries by producing plenty of heat in a short time. Furthermore, without a short circuit, a large amount of dead lithium with a high specific surface area generates during cycles, covering the surface of lithium metal anode. Though it is called dead lithium, yet there is still fresh lithium in dead lithium. Fresh lithium is wrapped by SEI and isolated from current collector, forming dead lithium. Dead lithium is usually powdered and has a large surface area. If the SEI of dead lithium is cracked by the increased temperature or other factors, plenty of fresh lithium in dead lithium inevitably reacts with electrolytes and generates much heat to further improve the internal temperature. The existence of dead lithium is unique in LMBs. Dead lithium may act as a new but strong contributor for the rapid rise of internal temperature at stage I of thermal production and thus raises the possibility of the final thermal runaway.

Electrolytes are involved into the three stages of the thermal runaway process and act as thermal transmitter. Electrolytes receive heat and then decompose. The decomposition of electrolytes further produces more heat and even high reactive species. Improving the thermal stability and nonflammability of electrolyte can suppress the parasitic reactions initiated by heat and decrease the further production of heat, thus interrupting the process of thermal runaway. Even the thermal runaway occurs, the nonflammable electrolyte cannot become the fuel of fire.

## 3 Evaluating Thermal Stability and Nonflammability

Evaluating the thermal stability and nonflammability of solvents, Li salts, and electrolytes is of great significance for understanding their thermal behaviors and working mechanism in suppressing thermal runaway, thus contributing to further electrolyte design. Some methods are briefly summarized in this section to evaluate the thermal stability and nonflammability qualitatively or quantitatively.

### 3.1 Differential Scanning Calorimeter

**Figure 2:**
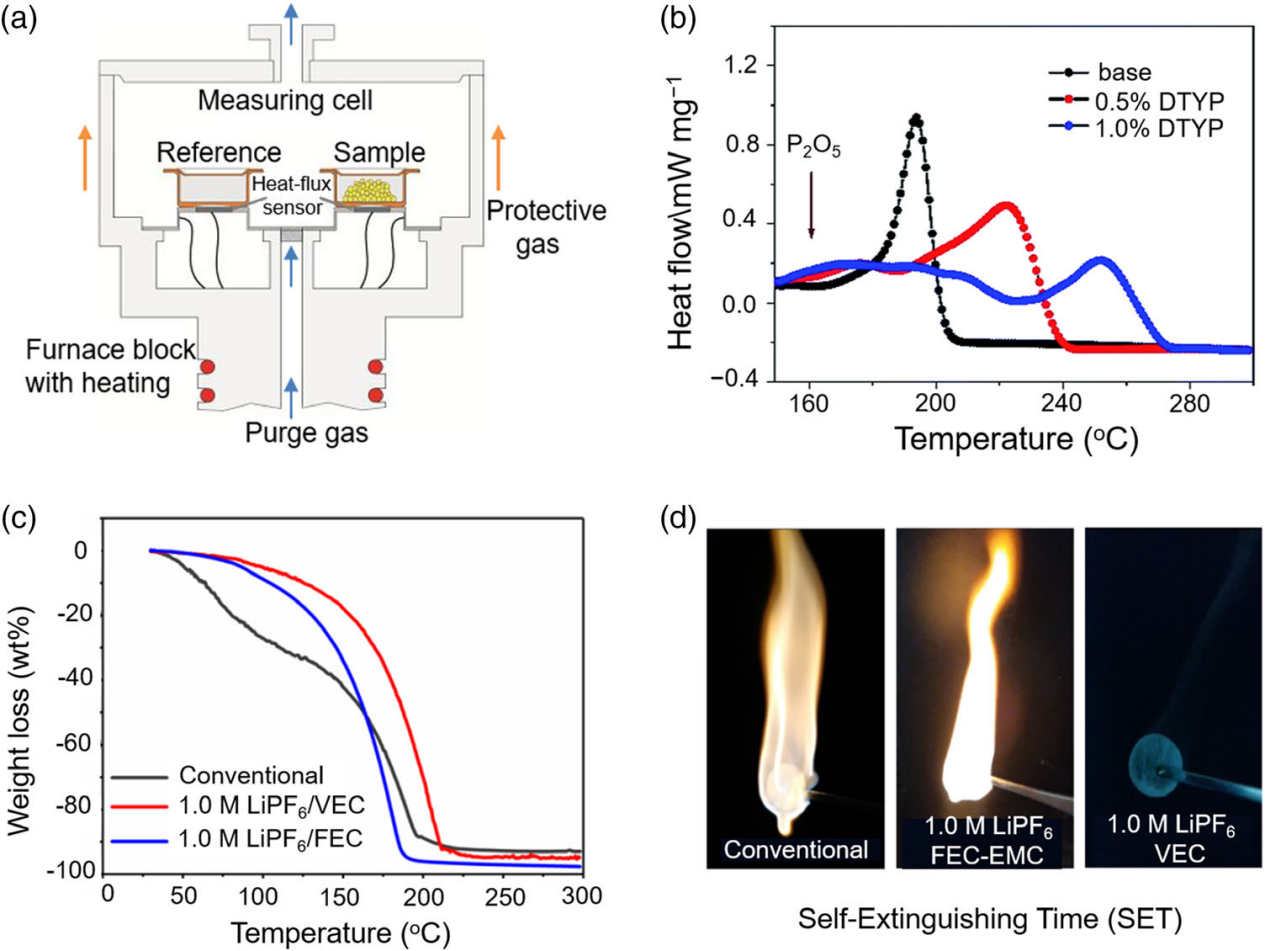
a) The schematic diagram of the DSC device. b) DSC curves of different electrolytes. Reproduced with permission.^[^ ^65^ ^]^ Copyright 2018, Royal Society of Chemistry. c) TGA curves of different electrolytes and d) SET tests of different electrolytes. c,d) Reproduced with permission.^[^ ^66^ ^]^ Copyright 2020, Elsevier.

Differential scanning calorimeter (DSC) is a thermoanalytical technique and it measures the difference in the amount of heat required for increasing the temperature of a test and reference sample with the change of temperature (**Figure** **2**a). Throughout the experiment, the sample and reference samples are kept at almost the same temperature and the temperature of the sample holder increases linearly with time. The heat capacity of the reference sample within the tested temperature range should be clearly defined.

The number, height, location, symmetry, and peak area of the differential thermal peaks can be clearly seen from DSC spectra. The number of peak indicates the properties of physical and chemical changes of the substance. The size and direction of the peak represent the magnitude and sign of the thermal effect. For example, the larger peak area represents that the more heat is absorbed and released. The peak tip upward proves that the reaction is exothermic. The position of the peak indicates the transformation temperature of the substance. As shown in Figure 2b, base electrolyte has a significant thermal decomposition at 186 °C.^[^ ^65^ ^]^ The direction of the peak is upward, indicating that the decomposition reaction of the electrolyte is an exothermic reaction. When the flame‐retardant additives are added to the base electrolyte, the exothermic temperature of the electrolyte can be increased to 223 and 252 °C. Although DSC can accurately measure the temperature and the amount of heat absorption and release of the electrolyte, yet it cannot directly identify what the concrete decomposition reaction is. In addition, the electrolyte is easy to volatilize during test, disturbing the accuracy of the test, which attention should be paid.

### 3.2 Thermogravimetric Analysis

Thermogravimetric analysis (TGA) refers to a thermal analysis technique that measures the relationship between the mass of the sample to be tested and the temperature change under program control temperature. TGA can measure the thermal stability and composition of a substance by testing the weight loss of a sample under heating. The quantitative TGA method can accurately measure the mass change and the rate of mass change of the substance, which affords to presume the decomposition mechanism of the substance.

From the electrolyte level, it is used to study the thermal stability of the electrolyte. Figure 2c shows the TGA results of different electrolytes.^[^ ^66^ ^]^ When heated to 150 °C, common electrolytes (1.0 m LiPF_6_ in EC/EMC) lost about 42 wt% mass due to solvent evaporation, which can increase the internal pressure and potential safety hazards of the battery. On the contrary, concentrated electrolytes (1.0 m LiPF_6_ in vinylethylene carbonate, VEC) show negligible volatility at 150 °C because the boiling point of EMC is much lower than that of VEC (107 vs 237 °C). TGA can judge the temperature range where the electrolyte is relatively stable through weight loss, providing guidance for the design of high‐safe electrolytes. Similarly, TGA cannot provide concrete decomposition reactions. However, the results of TGA, especially the change in the mass, provide fundamental information for inferring the decomposition reactions. Since the TGA is a general test conducted in an open system, the liquid electrolyte will inevitably volatilize, which may cause large deviations in the test results.

### 3.3 Limiting Oxygen Index

Limiting oxygen index (LOI) is the minimum O_2_ concentration (expressed as a percentage) that supports the combustion of a material. The measurement is performed by passing a mixture of O_2_ and N_2_ through the burning sample and reducing the O_2_ content until it reaches a critical level. It is an index that characterizes the combustion behavior of a material. The LOI method is as follows. First, a specific volume of sample is injected into the combustion tube, which contains a mixture of O_2_ and N_2_. Second, when the top of sample is ignited, the O_2_ concentration in the mixed gas stream will continue to decrease until the flame goes out. Finally, the minimum O_2_ concentration that supports the combustion of the material can be measured. The measured result is expressed as the value of the volume percentage occupied by O_2_. The high O_2_ index proves that the material is not easy to burn. According to the LOI, the flammability of materials can be divided into three categories: 1) LOI < 21% indicates that the material is highly flammable. 2) LOI > 28% indicates that the material is nonflammable; 3) the material is considered retarded when LOI is between aforementioned two values.^[^ ^67^ ^]^ Therefore, the flame retardancy of the electrolyte can be quantitatively described using LOI test method.

### 3.4 Self‐Extinguishing Time

The self‐extinguishing time (SET) refers to the extinguishing time after leaving the fire source (Figure 2d).^[^ ^66^ ^]^ The SET test of a certain mass of electrolyte can quantitatively determine the flammability of the electrolyte. According to the value of SET, flammability can be divided into three categories in detail: 1) flammable when SET > 20 s g^−1^; 2) nonflammable when SET < 6 s g^−1^; 3) retarded when SET is between aforementioned two values.^[^ ^68^ ^]^ However, the degree of linearity between SET and the mass of electrolyte should be taken care. In addition, there are human errors in manual SET, such as the judgment of fire‐fighting time. Therefore, the unified criteria should be defined rigorously.

### 3.5 Accelerating Rate Calorimeter

**Figure 3:**
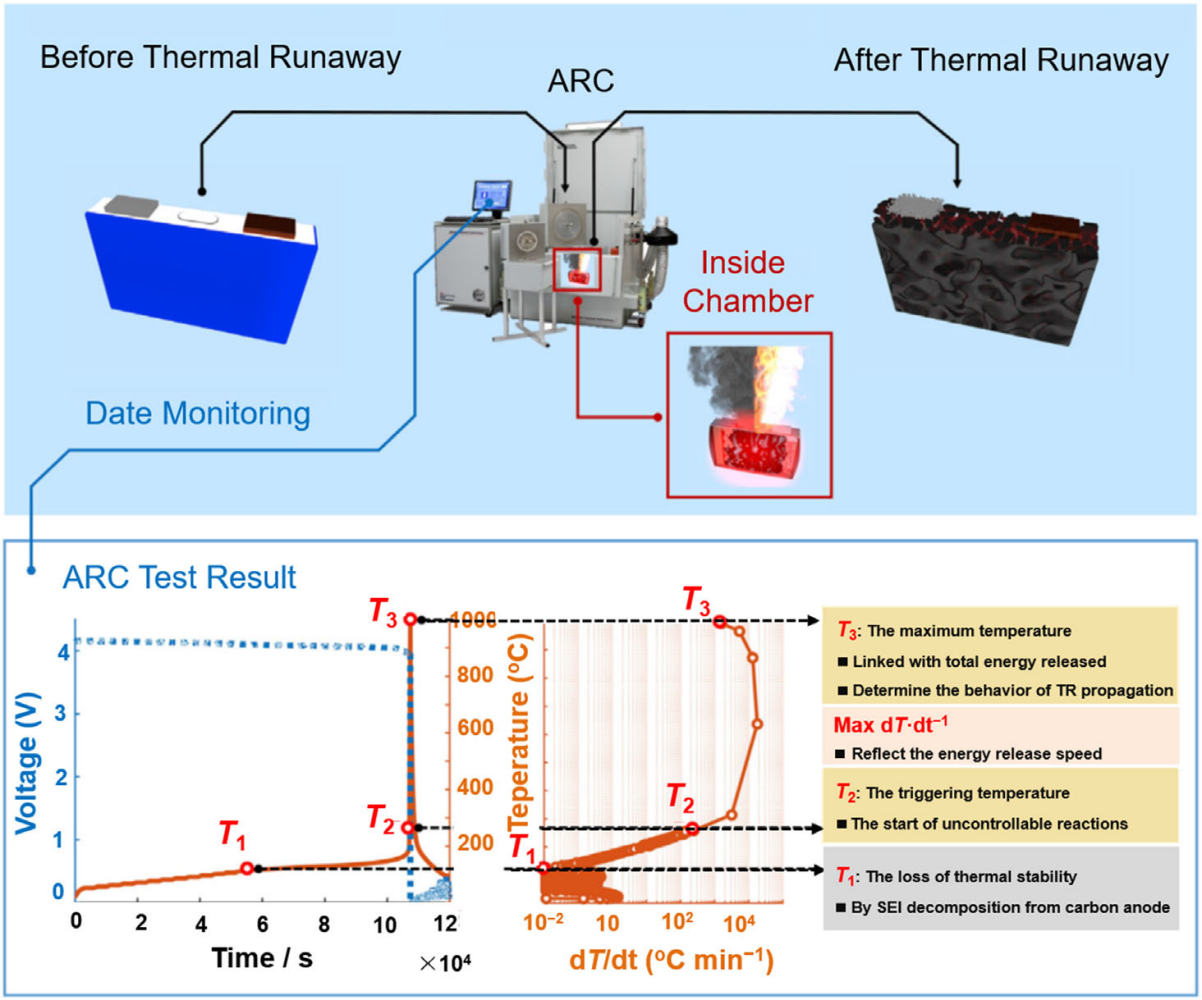
The testing schematic diagram and experimental results of ARC. Upper panel: the schemes of test process of ARC. Down panel: the test results of ARC, including the voltage—time curves, temperature–d*T*/d*t* curves and the corresponding illustration of *T*_1_, *T*_2_, and *T*_3_. Reproduced with permission.^[^ ^71^ ^]^ Copyright 2020, Elsevier.

The aforementioned test methods are mainly used to evaluate the thermal stability and nonflammability of solvents, Li salts, or electrolytes. However, the thermal stability and nonflammability of electrolytes in a practical battery must be considered. At present, the most representative method of battery safety testing is accelerating rate calorimeter (ARC). ARC is a test method that can provide adiabatic and calorimetric data in a safe experimental environment (**Figure** **3**).^[^ 69, 70 ^]^ The test principle is to use ARC to heat the battery, which can record the temperature change in the battery during thermal runaway process. ARC uses adiabatic kinetics to analyze some thermodynamic data, such as activation energy, reaction order, frequency factor, adiabatic temperature rise, and reaction heat. These data can be used to scale up and calculate the thermal hazard under real conditions. ARC can provide an adiabatic test environment that focuses on measuring heat generation and excludes the influence of heat dissipation to the environment. The specific process of ARC operation is as follows. The batteries are placed inside the ARC chamber, where it is heated to the state of thermal runaway. Once the ARC system detects that the test sample generates significant heat, it will follow the temperature of the test sample to provide an adiabatic test environment. ARC records signals, such as temperature and voltage, which are further used to analyze the thermal runaway mechanism. The thermal runaway state of the battery conforms to three characteristic temperatures (*T*_1_, *T*_2_, and *T*_3_).^[^ ^71^ ^]^ *T*_1_ represents the starting temperature of abnormal heat, indicating that the ARC system has detected significant heat from the side reactions inside the battery. *T*_2_ is the trigger temperature for battery thermal runaway. *T*_3_ is the highest temperature in thermal runaway. *T*_1_ and *T*_2_ are closely related to the stability of the electrolyte. *T*_1_ represents the temperature at which SEI starts to decompose. The heat generated by the decomposition and regeneration of SEI will cause the electrolyte to decompose, leading to heat accumulation inside the battery. As the internal temperature of the battery continues to rise, the separator is heated to shrink, which will cause the direct contact of anode and cathode, that is, internal short circuit in a battery. The internal short circuit in the batteries will produce much more heat according to Joule's law, which will improve *T*_1_ to *T*_2_. The effect of the thermal stability and nonflammability of the electrolyte is inferred by the temperature change in the ARC test, providing fundamental reference for the design of the thermal stable and nonflammable electrolytes.

The characterization methods for the thermal stability and nonflammability of electrolytes are summarized in **Table** **2**. It is obvious that each characterization method has its own advantages and disadvantages. Various test methods are suggested to be combined to comprehensively evaluate the thermal stability and nonflammability of the tested electrolyte.

**Table 2:** Comparison of test methods for the thermal stability and nonflammability of electrolytes

Characterization methods	DSC	TGA	LOI	SET	ARC
Characterization	Thermal stability	Thermal stability	Flammability	Flammability	Battery safety
Test accuracy	Good	Medium	Good	Medium	Good
Quality of test sample	Little	Little	Many	Medium	many

### 3.6 Free Radical Trapping Mechanism

**Figure 4:**
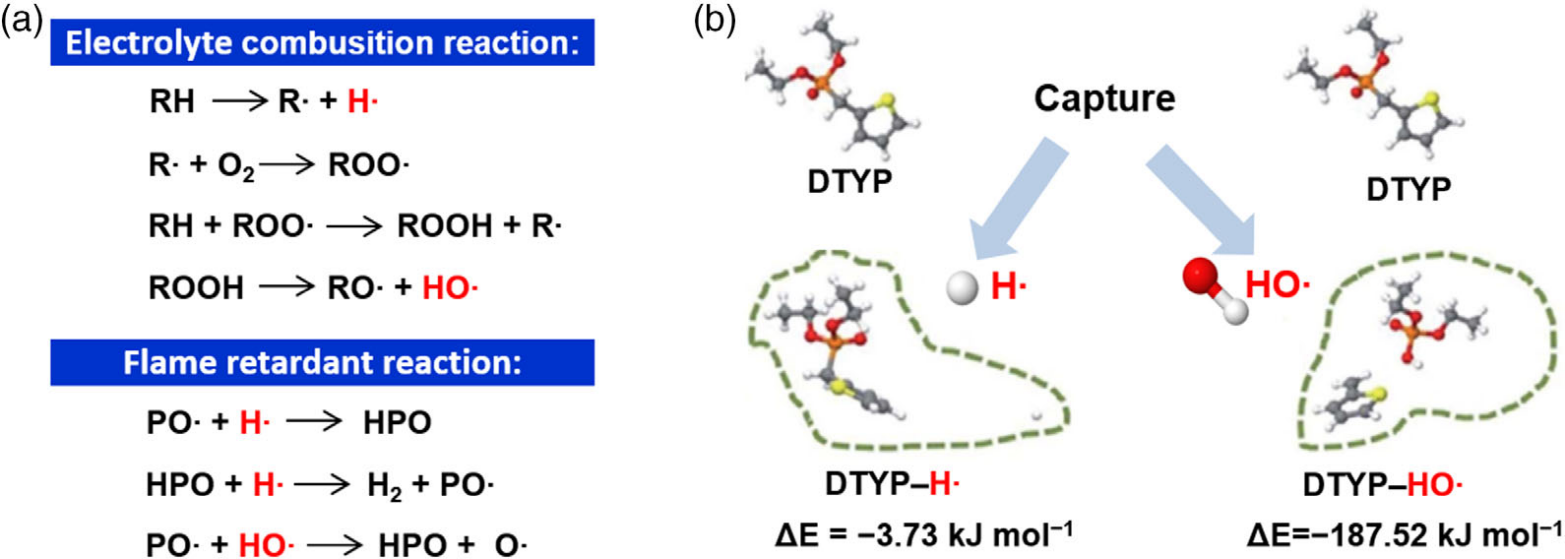
a) Interpretation of working mechanism of flame retardant in electrolyte system: electrolyte combustion reaction and flame‐retardant reaction. Reproduced with permission.^[^ ^72^ ^]^ Copyright 2018, Elsevier. b) Free radical capture mechanism in electrolyte. Reproduced with permission.^[^ ^65^ ^]^ Copyright 2018, Royal Society of Chemistry.

When a battery is misused or abused, the temperature inside the battery rises, followed by a series of continuous exothermic processes, inducing the heat accumulation in the battery. If the heat accumulation is severe, electrolytes undergo thermal decomposition, which can generate a large amount of hydrogen (H·) and hydroxide radicals (HO·). A polymerization reaction of radicals occurs between the aforementioned free radicals to form O_2_ and H_2_, which can bring high safety risks to the battery. Understanding the formation, polymerization, and capture mechanisms of free radicals is necessary to improve the thermal stability and nonflammability of electrolytes. Taking a more classic phosphate flame retardant as an example, its free radical scavenging mechanism is shown in **Figure** **4**a.^[^ ^72^ ^]^ First, the phosphorus flame retardant is heated and evaporated. The vapor formed can then generate P‐containing free radicals, such as phosphorous oxygen radicals (PO·). The PO· can effectively scavenge H· and HO·, thereby terminating free radical reactions and hindering flammability. Theoretical calculations are used to simulate the trapping mechanism of free radicals, which can provide rational guidance for the design of flame retardants. The density functional theory (DFT) calculation can be used to quantitatively describe the ability of flame‐retardant molecular to scavenge harmful radicals (Figure 4b).^[^ ^65^ ^]^ The low binding energy between flame‐retardant molecules and harmful free radicals, indicating that the flame‐retardant molecular has a stronger ability to capture harmful free radicals.

## 4 Improving Thermal Stability and Nonflammability

Although organic liquid electrolytes exhibit high ionic conductivity and wide electrochemical window, the thermal stability and nonflammability of liquid electrolytes must be improved to circumvent thermal runaway and even fire hazards or catastrophic explosions. In this section, some research progress of thermal stable and nonflammable electrolytes are summarized according to the classification of electrolytes. The thermal stable and nonflammable electrolytes in LMBs mainly include the following categories: phosphate ester‐based, all‐fluorinated, cyclic carbonate‐based, ionic liquids, and solid electrolytes.

### 4.1 Phosphate Ester‐Based Electrolytes

**Figure 5:**
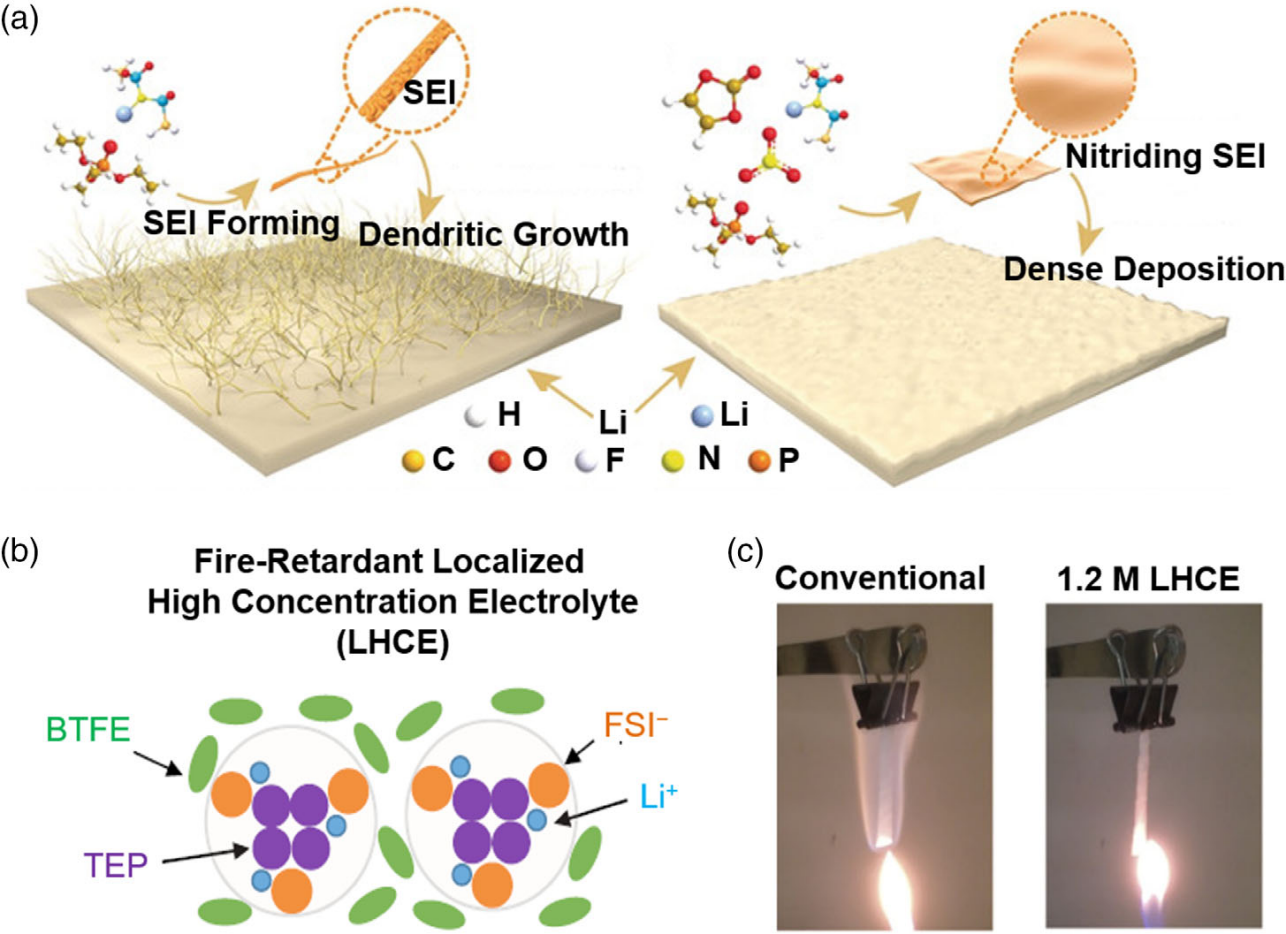
Nonflammable phosphate‐based electrolytes for LMBs. a) Schematic illustration of TEP‐based electrolyte for LMBs. Reproduced with permission.^[^ ^79^ ^]^ Copyright 2019, Wiley‐VCH. b) Schematic illustration of flame‐retardant LHCE (LiFSI‐1.33TEP‐3BTFE) and c) photographs of ignition tests of glass fibers saturated with the conventional electrolyte and LHCE. Reproduced with permission.^[^ ^81^ ^]^ Copyright 2018, Elsevier.

When the battery is misused or abused to generate heat accumulation, some organic solvents can thermally decompose to form H· and HO·, which further increases the safety risks of the battery. The phosphate esters have strong free radical scavenging ability and can block the chain reaction of harmful free radicals. In fact, as the internal temperature in battery increases, the phosphate esters can decompose and form PO·. PO· can further react with H· and HO· to terminate the chain reaction, thereby improving the nonflammability of the electrolyte. In addition, the low toxicity and low cost of phosphate esters are conducive to industrial applications. Currently, commonly used phosphate ester molecules include, trimethyl phosphate (TMP), triethyl phosphate (TEP), and so on.^[^ 73, 74, 75, 76, 77 ^]^ Since the nonflammable phosphate esters cannot form a stable SEI on the surface of lithium metal anode, which will cause the phosphate esters to continuously decompose and deteriorate the cycle performance of LMBs.^[^ ^78^ ^]^ Therefore, the prerequisite for the applications of phosphate esters electrolytes is to construct a stable SEI on the surface of lithium metal anode. Adding electrolyte additives, which can be reduced preferentially, to the phosphate‐based electrolyte is considered as an economical and effective strategy. Guo and coworkers introduced electrolyte additives, vinylene carbonate (VC) and lithium nitrate (LiNO_3_), into 1.0 m lithium bis(trifluoromethanesulfonyl)imide (LiTFSI) in TEP‐based electrolytes (**Figure** **5**a).^[^ ^79^ ^]^ The addition of LiNO_3_ additive facilitates the construction of nitriding interface on the surface of metallic lithium metal. Li | Li cell can achieve a stable cycling performance without dendrite growth. The additive‐containing electrolytes not only significantly enhance the long‐term cycling performance of LMBs but also guarantee the nonflammability. Due to the relatively low amount of electrolyte additives, however, constant consumption during cycle is inevitable, and a stable SEI cannot be formed continuously to protect lithium metal anode. The reason for the aforementioned situation is that there are many free phosphate ester molecules in low‐concentration electrolytes. The metallic lithium will continue to react with free phosphate ester solvents, leading to the consumption of active lithium and deteriorating the interface stability of lithium metal anode. Compared with low‐concentration phosphate ester electrolytes, high‐concentration phosphate ester electrolytes have fewer free solvent molecules. High‐concentration electrolytes recruit many phosphate ester solvents into the solvation sheath of lithium ions, resulting in a significant reduction in the number of free phosphate ester solvent molecules. Cao, Xiao, Liu, and coworkers designed a highly concentrated phosphate ester electrolyte consisted of LiFSI:TEP in a molar ratio of 1:2 with LiBOB and fluoroethylene carbonate (FEC) as electrolyte additives.^[^ ^80^ ^]^ With a high salt‐to‐solvent molar ratio (1:2), the phosphate ester molecules are mostly coordinated in the primary solvation shell of lithium ions, effectively suppressing the reactivity of nonflammable phosphate ester electrolytes toward lithium metal anode. The nail penetration test can evaluate the safety of nonflammable electrolytes in the battery. The battery with highly concentrated phosphate ester electrolytes remained intact during nail penetration, whereas the battery with common carbonate electrolytes cracked and exploded into flames. Therefore, a battery using TEP‐based electrolytes exhibits higher intrinsic safety than the battery using common carbonate electrolytes. Due to the high viscosity and high cost of high‐concentration electrolytes, it is not conducive to large‐scale industrial applications. To overcome the aforementioned issues, Zhang and coworkers proposed that hydrofluoroether as a cosolvent can be introduced to dilute high‐concentration phosphate ester electrolytes (1.2 m LiFSI in TEP), forming a localized high‐concentration electrolyte (LHCE, Figure 5b).^[^ ^81^ ^]^ The advantage of LHCE is ascribed to the well‐reserved Li^+^—FSI^−^—TEP solvation structure which is much like that in high‐concentration electrolytes, as well as the enhanced interface protection. LHCE not only significantly enhances the cycling performance of Li | NMC622 batteries but also guarantees the nonflammability of electrolyte. 1.0 m LiPF_6_ in EC/EMC electrolytes are highly flammable (Figure 5c). In contrast, 1.2 m LHCE cannot be ignited, which can significantly reduce the safety hazards of LMBs.

### 4.2 All‐Fluorinated Electrolytes

Fluorinated solvents and Li salts receive rising attention to improve the safety level of liquid electrolytes due to their high flash point, high thermal stability, and nonflammability.^[^ 82, 83 ^]^ The high thermal stability of fluorinated solvents is determined by its molecular structure. The C—F bond energy in fluorinated solvents is much higher than C—H bond in nonfluorinated solvents (105.4 vs 98.8 kcal mol^−1^).^[^ ^64^ ^]^ In addition, fluorinated solvents will produce fluorine radicals (F·) at high temperature, and it has strong ability to quench H· and OH· formed by the decomposition of organic solvents, effectively terminating the chain reactions induced by harmful free radicals and improving the nonflammability of the electrolytes.

To improve the nonflammability and thermal stability of the electrolytes, Amine, Xu, Wang, and coworkers reported an all‐fluorinated electrolyte composed of 1.0 m LiPF_6_ dissolved in a mixture of FEC, 3,3,3‐fluoroethylmethyl carbonate (FEMC), and 1,1,2,2‐tetrafluoroethyl‐2′,2′,2′‐trifluoroethyl ether (HFE), which exhibited nonflammability.^[^ ^84^ ^]^ The all‐fluorinated electrolyte constructed the interphases with high LiF content on both anode and cathode, thereby significantly improving the cycle performance of LMBs. Due to the limited types of nonflammable fluorinated solvent currently available, such as FEC, HFE, and BTFE, it is necessary to develop alternative fluorinated solvents with high thermal stability and nonflammability. Recently, Cui, Bao, and coworkers reported a novel fluorinated ether, fluorinated 1,4‐dimethoxylbutane (FDMB), which was synthesized by selectively functionalizing ether backbones with –CF_2_–groups.^[^ ^85^ ^]^ This electrolyte possesses Li–F binding and a high anion/solvent ratio in the solvation sheath of lithium ions, leading to stable interface compatibility with lithium metal anode. 1.0 m LiFSI/FDMB electrolytes endowed lithium metal anode with a thin interphase (≈6 nm), which was observed by cryogenic transmission electron microscopy (cryo‐TEM).

### 4.3 Nonflammable Cyclic Carbonate‐Based Electrolytes

The main cyclic carbonates in the electrolytes are EC and PC. In general, cyclic carbonates have high flash points. For example, the flash points of EC and PC are 160 and 135 °C, respectively, which will ensure the thermal stability of electrolytes.^[^ 86, 87, 88 ^]^ Borodin, Lee, and coworkers reported a cyclic carbonate electrolyte, 4.0 m LiTFSI in PC with FEC additives.^[^ ^89^ ^]^ To verify the high flame retardancy of the electrolytes, lithium metal, NCM811 cathodes, and even polyethylene separators have been preimpregnated with 4.0 m LiTFSI in PC containing FEC additives, which cannot be ignited when exposed to flame. At the same time, the thermal stability of the electrolytes has also been well verified. To further evaluate the safety behaviors of lithium metal pouch batteries under the fully charged state (4.2 V), a 500 mAh pouch cell was stored at a high temperature at 130 °C to further observe the gassing behaviors. The pouch cell containing 1.0 m LiTFSI in DOL/DME electrolyte produced obvious gassing at high temperature. In contrast, a pouch cell containing 4.0 m LiTFSI in PC with FEC additives maintained its voltage above 4.0 V without dimensional expansion or deformation. Therefore, 4.0 m LiTFSI in PC with FEC additives has high thermal stability and nonflammability, which significantly reduces the safety hazards of pouch cells. Li, Xu, and coworkers reported a cyclic carbonate electrolyte that consists of 1.0 m LiPF_6_ dissolved in vinylethylene carbonate (VEC).^[^ ^66^ ^]^ The SET experiment illustrates that the electrolyte aerosol released by 1.0 m LiPF_6_ in EC/EMC electrolyte is highly flammable, and the SET is 51 ± 2 s g^−1^. In contrast, the SET of 1.0 m LiPF_6_ in VEC is nearly zero, indicating that the electrolyte is nonflammable. TGA is used to measure the thermal stability of the electrolytes. When heated to 150 °C, 1.0 m LiPF_6_ in EC/EMC electrolyte loses about 42 wt% of its mass due to solvent evaporation, which may accumulate battery pressure sufficient to damage the battery's sealing. In comparison, 1.0 m LiPF_6_ in VEC electrolyte shows negligible volatility. The mass loss is only 18% at 150 °C. This resistance to evaporation may result from the high boiling point of VEC (237 °C) compared with EMC (107 °C). In addition, the safety of pouch cells was evaluated by puncturing test. During the puncturing, the pouch cell containing 1.0 m LiPF_6_ in EC/EMC electrolyte will catch fire obviously. In contrast, pouch cells containing 1.0 m LiPF_6_ in VEC electrolytes do not emit fire or smoke.

### 4.4 Ionic Liquid Electrolytes

**Figure 6:**
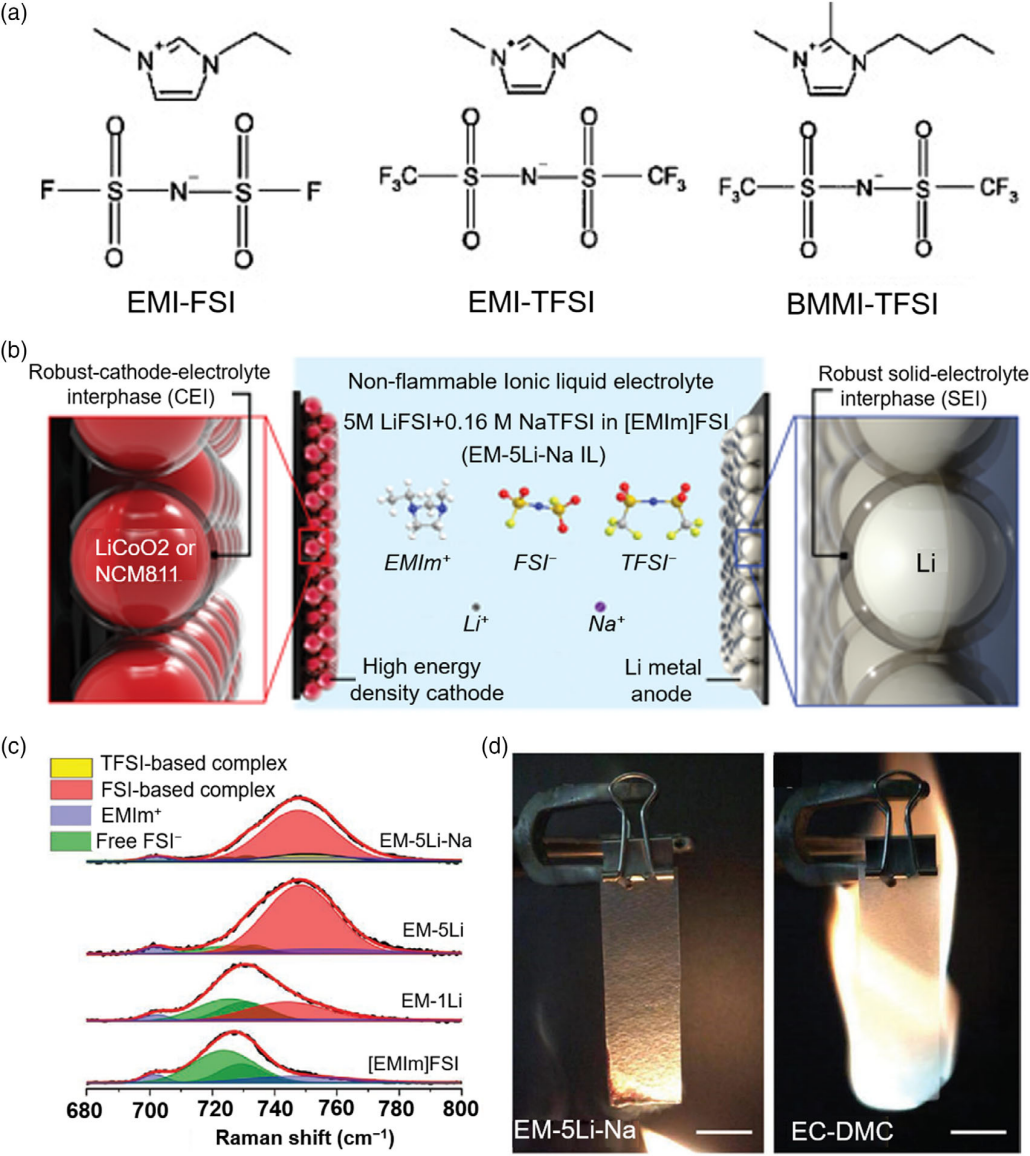
Nonflammable ionic liquid‐based electrolytes for LMBs. a) Structures of some types of ionic liquid. Reproduced with permission.^[^ ^90^ ^]^ Copyright 2009, Elsevier. b) Schematic illustration of the battery configuration and electrolyte composition of ionic liquid‐based electrolytes. c) Raman spectra of different compositions of ionic liquid‐based electrolytes and d) flammability tests of ionic liquid‐based electrolytes and conventional organic electrolytes. b–d) Reproduced with permission.^[^ ^96^ ^]^ Copyright 2020, Wiley‐VCH.

The ionic liquid usually refers to the liquid salt at room temperature composed of cations and anions completely. Their representative molecular structures are shown in **Figure** **6**a.^[^ ^90^ ^]^ The ionic liquid has a high boiling point and a flash point to improve the thermal stability and nonflammability of the electrolytes. Since the ionic liquid cannot form a stable SEI on the surface of lithium metal anode, it is difficult to be used as an electrolyte solvent. Therefore, high interface stability between ionic liquid and lithium metal anode is a prerequisite for its applications in LMBs. At present, researches on nonflammable ionic liquid electrolytes in LMBs are mainly focused on increasing the salt concentration.^[^ 91, 92, 93, 94, 95 ^]^ Dai and coworkers reported nonflammable ionic liquid electrolytes composed of 1‐ethyl‐3‐methylimidazolium (EMIm) cations and high‐concentration LiFSI with sodium bis(trifluoromethanesulfonyl)imide (NaTFSI) as an additive. The ionic liquid electrolyte participates in the formation of hybrid interphases and contributes to dense lithium deposition (Figure 6b).^[^ ^96^ ^]^ This highly stable lithium metal anode interface is closely related to the solvation structure in highly concentrated ionic liquid. The Raman spectra proves that the number of free FSI anions decreases in the high‐concentration ionic liquid electrolytes and FSI anions participate into the solvation of Li ions (Figure 6c). The nonflammability of ionic liquid electrolytes was verified in comparison to 1.0 m LiPF_6_ in EC/DMC electrolytes (Figure 6d).

### 4.5 Solid‐State Electrolytes

Solid‐state electrolytes are developed to avoid the battery failure induced by internal short circuits or leak.^[^ 97, 98, 99, 100, 101, 102, 103, 104, 105 ^]^ Solid‐state electrolytes include quasi and all‐solid‐state electrolytes. Although all‐solid‐state electrolytes theoretically achieve supreme electrolyte safety due to the abandonment of flammable liquid electrolytes, yet it suffers tough interfacial issues and low ionic conductivity. Quasisolid‐state electrolytes combine the advantages of liquid‐ and solid‐state electrolytes, including good thermal stability, low interfacial resistance, and high ionic conductivity.

**Figure 7:**
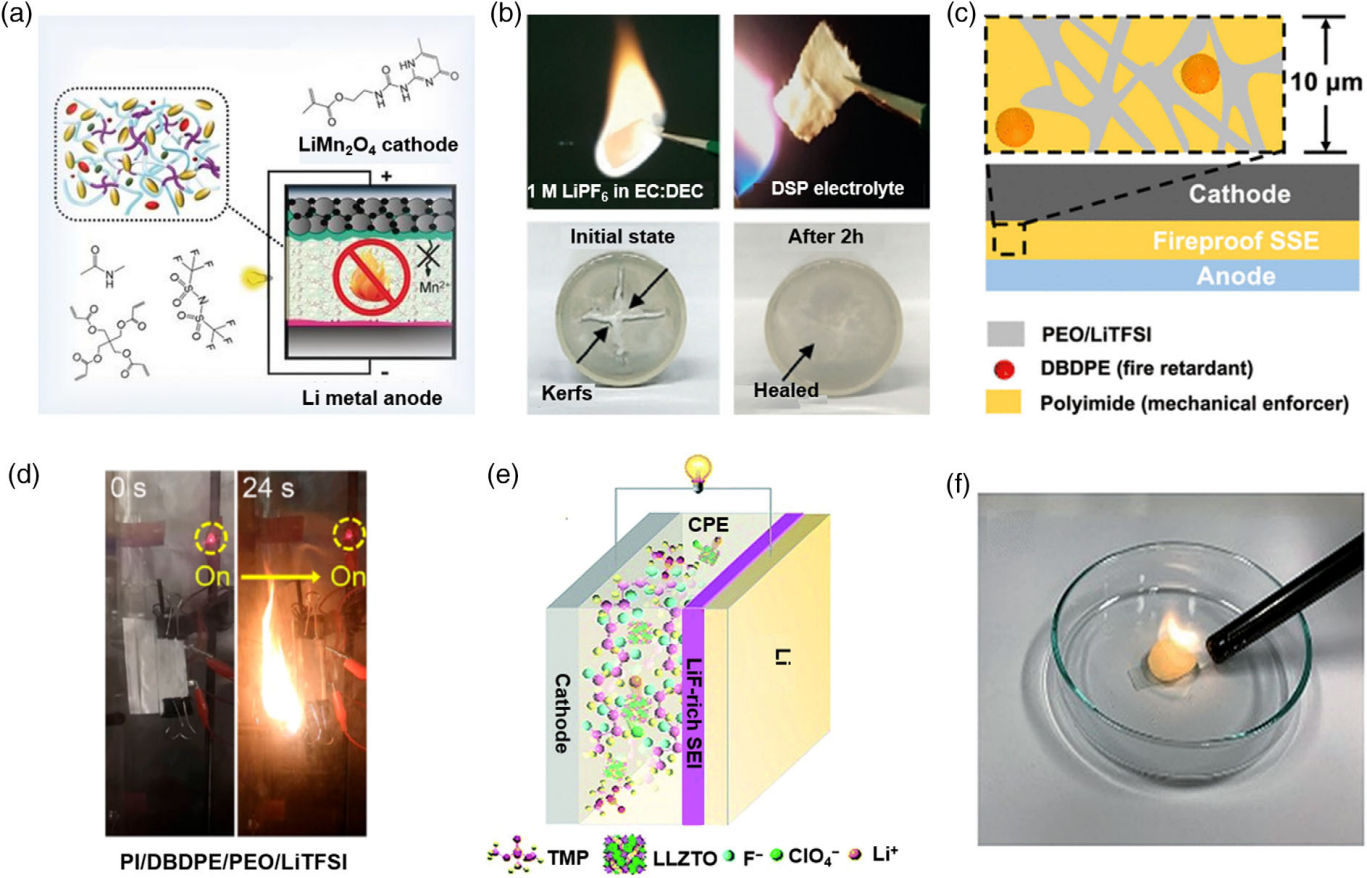
Nonflammable solid‐state electrolytes for LMBs. a) Solid–liquid equilibrium of NMAc:LiTFSI binary mixture as a function of LiTFSI mole fraction and b) safety test of DES. a,b) Reproduced with permission.^[^ ^106^ ^]^ Copyright 2020, Wiley‐VCH. c) Design principles of the fireproof and lightweight polymer solid‐state electrolyte and d) flame abuse test of batteries with PI/DBDPE/PEO/LiTFSI as electrolyte, respectively. c,d) Reproduced with permission.^[^ ^109^ ^]^ Copyright 2020, American Chemical Society. e) Schematic showing a solid‐state electrolyte composed of PVDF, LLZTO, TMP, and LiClO_4_ and f) evaluation of the flammability of solid‐state electrolyte. e,f) Reproduced with permission.^[^ ^110^ ^]^ Copyright 2019, Royal Society of Chemistry.

Zhou, Li, Wang, and coworkers reported a deep eutectic solvent (DES)‐based quasisolid‐state electrolyte.^[^ ^106^ ^]^ This electrolyte was fabricated in a facile method by in situ copolymerization of pentaerythritol tetraacrylate (PETEA) and 2‐(3‐(6‐methyl‐4‐oxo‐1,4‐dihydropyrimidin‐2‐yl)ureido)ethyl methacrylate (UPyMA) monomers in DES‐based electrolytes containing FEC as an electrolyte additive (**Figure** **7**a). The highly nonflammable quasisolid‐state electrolytes can reduce potential safety hazards caused by electrolyte leakage (Figure 7b). Nitrile‐based quasisolid‐state electrolytes have high thermal stability and nonflammability. Cui, Dong, and coworkers developed a dual‐anion deep eutectic solution (D‐DES) based on succinonitrile (SN) and dual Li salts (LiTFSI and LiDFOB), and used it as the electrolyte in Li metal batteries.^[^ ^107^ ^]^ D‐DES displays the negligible weight loss at 150 °C and still retained about 77% of its pristine weight at 200 °C revealed by TGA curves. In addition, flammability test revealed that neither D‐DES electrolytes nor its surface vapor can be ignited by a lighter. Kang, He, and coworkers made a gel electrolyte by in situ polymerizing cyanoethyl polyvinyl alcohol (PVA‐CN) in a succinonitrile (SN) solvent.^[^ ^108^ ^]^ Compared with the highly flammable liquid electrolytes and the medium flammable gel polymer electrolytes, PVA–CN/SN gel electrolytes exhibit significantly lower flammability and are hardly ignited by flames. In addition, PVA–CN/SN gel electrolytes are negligibly volatile until a high temperature of 160 °C, which is mainly attributed to the inherent inertness of C≡N bonds.

Cui and coworkers reported the design of an ultralight weight and fire proof polymer solid‐state electrolyte. The solid‐state electrolyte is composed of a fire‐retardant additive (decabromodiphenyl ethane, DBDPE), a porous mechanic enforcer (polyimide, PI), an ionic conductive polymer electrolyte poly(ethyleneoxide) (PEO), and LiTFSI (Figure 7c).^[^ ^109^ ^]^ The light emitting diode (LED) bulb tested by the PI/DBDPE/PEO/LiTFSI pouch cell at 24 s was still as bright as that before ignition (Figure 7d). The test results show that solid‐state electrolyte is nonflammable, which improves the safety of LMBs. Ciucci and coworkers reported solid‐state electrolytes consisting of a poly(vinylidene fluoride) matrix, Li_6.4_La_3_Zr_1.4_Ta_0.6_O_12_ fillers, a LiClO_4_ salt, and a flame‐retardant TMP as a solvent (Figure 7e).^[^ ^110^ ^]^ In the ignition test, the solid‐state electrolyte exhibits high nonflammability (Figure 7f) which are detrimental to the functioning of LLZO‐based SSBs. Ciucci and coworkers reported all‐solid‐state electrolytes consisting of Ta‐doped‐LLZO (LLZTO) by employing a plastic crystal interlayer based on SN with a FEC additive.^[^ ^111^ ^]^ Compared with commonly used liquid electrolytes, all‐solid‐state electrolyte exhibits highly thermal stability. In addition, Ciucci and coworkers reported a nonflammable elastic quasisolid electrolyte based on poly(butyl acrylate) (PBA).^[^ ^112^ ^]^ The PBA‐based electrolytes exhibit nonflammability and thermally stability simultaneously.

**Figure 8:**
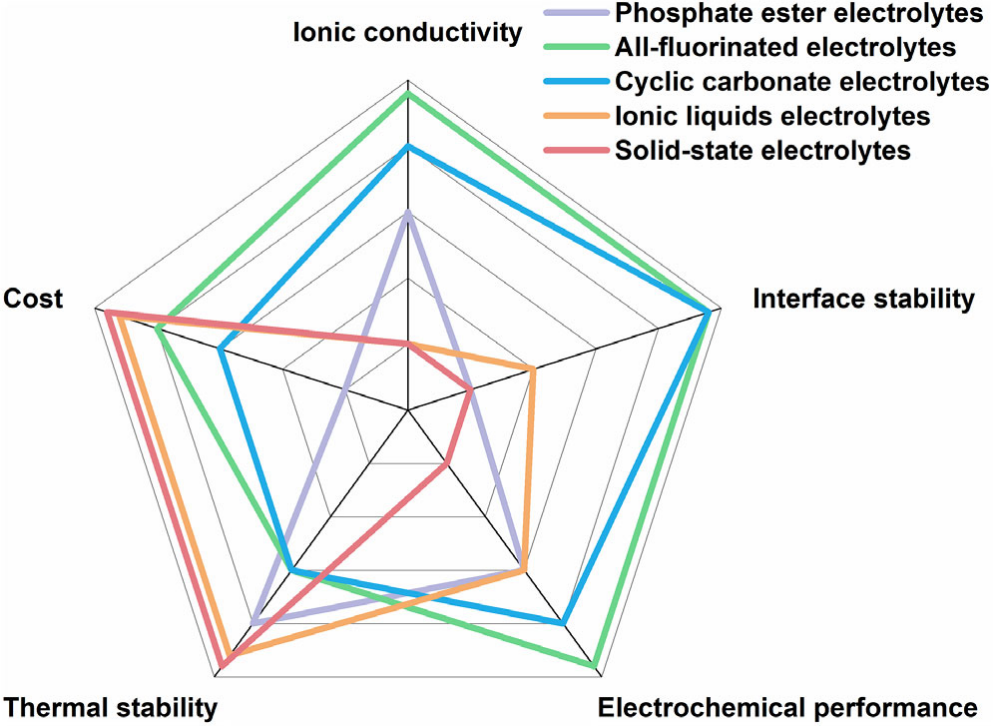
Comparison of the properties and performances of five nonflammable electrolytes.

The main characteristics of nonflammable electrolytes are listed in **Figure** **8**. In general, phosphate ester‐based, nitrile‐based, and ionic liquid electrolytes have attractive prospects with nonvolatility and high thermal stability. However, the common disadvantage of these electrolyte is that they cannot form a stable SEI on lithium metal anode, especially at a low salt concentration (1.0 m). Introducing electrolyte additives and improving the salt concentration can be used to improve the compatibility of the phosphate ester‐based electrolytes with lithium metal anode. In contrast, fluorinated solvents and some cyclic carbonate‐based electrolytes display relatively high interfacial compatibility with lithium metal anode, which is mainly due to their ability to decompose to form a stable SEI. However, these solvent molecules also have their own shortcomings. For example, FECs are easy to decompose and produce gas, which will bring potential hazards to the battery. In addition, the high cost of FEC is not conducive to large‐scale applications. Solid‐state electrolyte is regarded as one of the ultimate means to improve battery safety. However, solid‐state electrolyte also has its drawbacks, such as poor interface resistance and the stability against air, which hinders the process of solid‐state electrolyte to replace traditional liquid electrolytes in a short time. At this stage, fluorinated electrolytes display the most promising performance in terms of the balance between thermal stability and nonflammability and battery cycling performance. However, the thermal stability and nonflammability of fluorinated electrolytes should be further improved, especially at cell level. Furthermore, the weaknesses of fluorinated electrolytes, such as the gassing and cost, must be overcome when it comes to practical applications.

## 5 Conclusions and Outlook

LMB with high energy density is a promising next‐generation battery system, and the high safety is the precondition of practical applications of LMBs. However, liquid electrolytes with poor thermal stability and high flammability bring safety risks to LMBs by thermal runaway. The development of thermal stable and nonflammable electrolytes to solve the potential safety risks of liquid electrolytes is imperative. Although pioneering progress has achieved, yet the fundamental understandings of the thermal runaway process in LMBs and the rational design of thermal stable and nonflammable electrolytes are still scarce at this stage. Therefore, further research on thermal stable and nonflammable electrolytes is strongly required. 1) The thermal runaway process of LMBs. The thermal runaway process of LMBs is much different from LIBs due to the distinctive battery chemistry of anode, especially the effect of dead lithium on thermal runaway. The concrete thermal runaway process and the underlying reason should be identified first for rational improvement. Quantifying the heat generated in each step of thermal runaway process makes much sense for disclosing the evolution and simulations of thermal runaway process. 2) The compatibility between electrolyte and lithium metal anode. The thermal stable and nonflammable electrolytes generally display poor compatibility with lithium metal anode as reported, which significantly deteriorates the cycling performance of batteries. Constructing a stable SEI on the surface of lithium metal anode while employing thermal stable and nonflammable electrolytes is imperative for stable and highly safe LMBs, which is a challenged but significative task. The rational design of the solvation components and structure of lithium ions in electrolytes provides an effective method to achieve the aforementioned goal. 3) New thermal stable and nonflammable solvents. At present, the types of thermal stable and nonflammable organic solvents are limited. Therefore, the molecular design and synthesis of new solvents are required. The compatibility with lithium metal anode, thermal stability, nonflammability, cost, and environmental friendliness should be taken into consideration at the same time. The theoretical simulations can assist the molecular design. In addition, the flame‐retardant mechanisms of conventional flame‐retardant solvent molecules (e.g., TMP and TEP) are still not clear, and the fundamental flame‐retardant mechanisms need to be explored. 4) The risk of lithium salts. Lithium salt possibly brings safety risks to LMBs. Recently, although the solvent is nonflammable, the exothermic reduction reaction of lithium salts (LiFSI) can also trigger the thermal runaway of LIBs.^[^ ^113^ ^]^ Although the aforementioned test is in LIBs, the safety risks of lithium salts deserve addition in LMBs. Synergistically improving the thermal stability and nonflammability of lithium salts and solvents is necessary to enhance the safety of LMBs. 5) The safety at cell level. Electrolyte safety generally does not mean battery safety. Simply using thermal safety test methods (e.g., DSC, TGA, and SET) to evaluate the thermal stability and nonflammability of electrolyte itself cannot represent the safety of the electrolyte under the battery scale. The effect of thermal stable and nonflammable electrolyte on the safety of batteries should be evaluated in a practical battery for substantial progress. ARC and nail penetration tests are suggested to obtain the safety improvement at cell level.

Thermal stability and nonflammability of electrolyte are vital for improving the safety of LMBs. However, the fundamental understanding and design of thermal stable and nonflammable electrolytes in LMBs are still in its infancy and more researches are imperative for long‐cycling and high‐safety LMBs.

## Conflict of Interest

The authors declare no conflict of interest.
